# Correction: The interplay between eWOM information and purchase intention on social media: Through the lens of IAM and TAM theory

**DOI:** 10.1371/journal.pone.0314624

**Published:** 2024-11-27

**Authors:** Md. Atikur Rahaman, H. M. Kamrul Hassan, Ahmed Al Asheq, K. M. Anwarul Islam

There are errors in the Funding statement. The correct Funding statement is as follows: This study was supported by the School of Management, Jiujiang University and Center for Entrepreneurship & Innovation Research Society (grant number: 43120227).
