# Supplementary material for: The interplay between eWOM information and purchase intention on social media: Through the lens of IAM and TAM theory
Source: PLoS One. 2022 Sep 6;17(9):e0272926. doi: 10.1371/journal.pone.0272926 (PMC9447927; doi:10.1371/journal.pone.0272926)
Supplement: Attachment — Submitted filename: Responses to Reviewers_PONE-D-21-35683.docx [file pone.0272926.s002.docx]

**Responses to Reviewers (PONE-D-21-35683**)

“The Interplay between eWOM Information and Purchase In-tention on Social Media: Through the Lens of IAM and TAM Theory”

11 March 2022

Respected Reviewers,

Kindly find the below point to point responses to all reviewers’ comments

**Reviewer 1:**

| No. | Comments of Reviewer-01 | Our Responses |
| --- | --- | --- |
| 1. | Is the manuscript technically sound, and do the data support the conclusions?  The manuscript must describe a technically sound piece of scientific research with data that supports the conclusions. Experiments must have been conducted rigorously, with appropriate controls, replication, and sample sizes. The conclusions must be drawn appropriately based on the data presented.  Reviewer’s comment: Yes | Thank you so much for your positive compliment. |
| 2. | Has the statistical analysis been performed appropriately and rigorously?  Reviewer’s comment: Yes | Thank you so much for your positive feedback. |
| 3. | Have the authors made all data underlying the findings in their manuscript fully available?  The PLOS Data policy requires authors to make all data underlying the findings described in their manuscript fully available without restriction, with rare exception (please refer to the Data Availability Statement in the manuscript PDF file). The data should be provided as part of the manuscript or its supporting information, or deposited to a public repository. For example, in addition to summary statistics, the data points behind means, medians and variance measures should be available. If there are restrictions on publicly sharing data—e.g. participant privacy or use of data from a third party—those must be specified.  Reviewer’s comment: No | Thank you for your suggestion. Relevant data sets (1. Raw data file_csv file, 2. Measurement model_xlsx file, 3. Structural model__xlsx file) have been provided with the revised manuscript. |
| 4. | Is the manuscript presented in an intelligible fashion and written in standard English?  PLOS ONE does not copyedit accepted manuscripts, so the language in submitted articles must be clear, correct, and unambiguous. Any typographical or grammatical errors should be corrected at revision, so please note any specific errors here.  Reviewer’s comment: Yes | Thank you so much for your positive feedback. |
| 5. | This is a well-designed study with a solid theoretical foundation and reliable findings. | Thank you so much for your positive compliment. |
| 6. | The problem statement of this study is not convincing. The authors state that "aspects of eWOM information and online purchase intention on social media sites are not well explored in the literature”, but fail to explain in detail the novelty of this study. According to the search, various studies have been conducted to examine the characteristics of eWOM, or customer reviews, and also their impact on customer behavior. Furthermore, the cited literature supporting the necessarity is outdated (Cheung and Thadani, 2012). The authors should clearly explain how this study differs from previous studies. | Thank you for your valuable review comment. The study's problem statement, contribution, and research gap have been strengthened with recent citations (kindly refer to page 3 to 4, **yellow marked**).  In the marked yellow portion of page 3, **09 (nine) latest citations** are provided to enhance the quality of the paper. |
| 7. | The rest part of this study is well written, systematically presenting the theoretical background of the research and the relevant concepts. | Thank you so much for your positive feedback. |
| 8. | Also the research process is very scientific. However, I personally do not feel that the contribution of the research results is very significant.  Firstly, the study is not specific to a particular industry or related product, the adoption criteria for eWom may vary greatly with the product or industry;  secondly, the sample of the study is only limited to students. | The study’s contribution is exhibited in the page 3 & 4 (**yellow marked**).  The paper does not focus on any specific product category as this can be perceived as one of the limitations of this study (**please refer to page 22, yellow marked**).  Regarding the sampling, we’ve also considered this as our limitation as we drew samples only from the students (**please refer to page 22, blue colored**). |
| 9. | Overall, this is a well-designed study with excellent writing. The main limitation is that the contribution of this research is not very significant. | Thank you for your review feedback. The study’s contribution is exhibited in the page 3 & 4 (**yellow marked**). |

**Reviewer 2:**

| No. | Comments of Reviewer-02 | Our Responses |
| --- | --- | --- |
| 1. | Is the manuscript technically sound, and do the data support the conclusions?  The manuscript must describe a technically sound piece of scientific research with data that supports the conclusions. Experiments must have been conducted rigorously, with appropriate controls, replication, and sample sizes. The conclusions must be drawn appropriately based on the data presented.  Reviewer’s comment: Yes | Thank you so much for your positive compliment. |
| 2. | Has the statistical analysis been performed appropriately and rigorously?  Reviewer’s comment: Yes | Thank you so much for your positive feedback. |
| 3. | Have the authors made all data underlying the findings in their manuscript fully available?  The PLOS Data policy requires authors to make all data underlying the findings described in their manuscript fully available without restriction, with rare exception (please refer to the Data Availability Statement in the manuscript PDF file). The data should be provided as part of the manuscript or its supporting information, or deposited to a public repository. For example, in addition to summary statistics, the data points behind means, medians and variance measures should be available. If there are restrictions on publicly sharing data—e.g. participant privacy or use of data from a third party—those must be specified.  Reviewer’s comment: No | Thank you for your suggestion. Relevant data sets (1. Raw data file_csv file, 2. Measurement model_xlsx file, 3. Structural model__xlsx file) have been provided with the revised manuscript. |
| 4. | Is the manuscript presented in an intelligible fashion and written in standard English?  PLOS ONE does not copyedit accepted manuscripts, so the language in submitted articles must be clear, correct, and unambiguous. Any typographical or grammatical errors should be corrected at revision, so please note any specific errors here.  Reviewer’s comment: Yes | Thank you so much for your positive feedback. |
| 5. | This is a well-designed study with a solid theoretical foundation and reliable findings. | Thank you so much for your positive compliment. |
| 6. | Overall the paper was written in good English but with some mistakes. There are errors in citation, for e.g. D.-H. Park, H. Lee and some others. advised by (Hair Jr et al., 2021) to order?  Overall is a OK paper but not exciting. | Thank you for your suggestions. We have corrected all in-text citations throughout the manuscript (please refer to yellow marked in-text citations in page 6, 7, 12, 13.  The study's problem statement, contribution, and research gap have been strengthened with recent citations (kindly refer to page 3 to 4, **yellow marked**). In the marked yellow portion of page 3, **09 (nine) latest citations** are provided to enhance the quality of the paper. |

**NOTE:** For This study was supported by the School of Management, Jiujiang University, China. There is no grant number issued by the department. In regard to data availability statement, we’ve provided all necessary data files with the manuscript (please refer to 1. Raw data file_csv file, 2. Measurement model_xlsx file, 3. Structural model__xlsx file).

Kind Regards,

The Corresponding Author:

*Ahmed Al Asheq*

Lecturer

Department of Business Administration

World University of Bangladesh (WUB), Bangladesh.

Email: aasheq@business.wub.edu.bd

**Other Authors:**

Dr. Md. Atikur Rahaman (email: atik@jju.edu.cn)

H. M. Kamrul Hassan (email: kamrul@cu.ac.bd)
