# Supplementary material for: The interplay between eWOM information and purchase intention on social media: Through the lens of IAM and TAM theory
Source: PLoS One. 2022 Sep 6;17(9):e0272926. doi: 10.1371/journal.pone.0272926 (PMC9447927; doi:10.1371/journal.pone.0272926)
Supplement: Attachment — Submitted filename: Responses to Reviewers_PONE-D-21-35683R2.docx [file pone.0272926.s004.docx]

**Responses to Reviewers (PONE-D-21-35683R2**)

“The Interplay between eWOM Information and Purchase Intention on Social Media: Through the Lens of IAM and TAM Theory”

07 July 2022

Respected Reviewers,

Kindly find the below point to point responses to all reviewers’ comments

**Reviewer 1:**

| No. | Comments of Reviewer-01 | Our Responses |
| --- | --- | --- |
| 1. | If the authors have adequately addressed your comments raised in a previous round of review and you feel that this manuscript is now acceptable for publication, you may indicate that here to bypass the “Comments to the Author” section, enter your conflict of interest statement in the “Confidential to Editor” section, and submit your "Accept" recommendation.  Reviewer-1’s comment: All comments have been addressed. | Thank you so much for your positive compliment. |
| 2. | Is the manuscript technically sound, and do the data support the conclusions?  The manuscript must describe a technically sound piece of scientific research with data that supports the conclusions. Experiments must have been conducted rigorously, with appropriate controls, replication, and sample sizes. The conclusions must be drawn appropriately based on the data presented.  Reviewer-1’s comment: Yes | Many thanks for your positive feedback. |
| 3. | Has the statistical analysis been performed appropriately and rigorously?  Reviewer-1’s comment: I Don't Know | We have utilized deductive research approach as this method is suitable for empirical study (please refer to first para of “section 4. Materials and Method”, page 10). Our sample size is 432 which is statistically deemed as acceptable sample size for quantitatve study (Burns et al., 1995). We have described our development of survey instrument is reported in the 2^nd^ paragraph in page 11. We have also reported convergent and discriminant validity to verify construct reliability and validity (page 13-15).  Burns, A. C., Burns, K. R., & Bush, R. F. (1995, March). The SPSS Student Assistant: The Integration of a Statistical Analysis Program into a Marketing Research Textbook. *In Developments in Business Simulation and Experiential Learning: Proceedings of the Annual ABSEL conference (Vol. 22)*. |
| 4. | Have the authors made all data underlying the findings in their manuscript fully available?  Reviewer-1’s comment: Yes | Thank you so much for your positive compliment. |
| 5. | Is the manuscript presented in an intelligible fashion and written in standard English?  Reviewer-1’s comment: Yes | Thank you so much for your positive compliment. |
| 6. | Please use the space provided to explain your answers to the questions above. You may also include additional comments for the author, including concerns about dual publication, research ethics, or publication ethics. (Please upload your review as an attachment if it exceeds 20,000 characters).  Reviewer-1’s comment: (No Response) | Thank you. |

**Reviewer 2:**

| No. | Comments of Reviewer-02 | Our Responses |
| --- | --- | --- |
| 1. | If the authors have adequately addressed your comments raised in a previous round of review and you feel that this manuscript is now acceptable for publication, you may indicate that here to bypass the “Comments to the Author” section, enter your conflict of interest statement in the “Confidential to Editor” section, and submit your "Accept" recommendation.  Reviewer-2’s comment: (No Response) | Thank you. |
| 2. | Is the manuscript technically sound, and do the data support the conclusions?  The manuscript must describe a technically sound piece of scientific research with data that supports the conclusions. Experiments must have been conducted rigorously, with appropriate controls, replication, and sample sizes. The conclusions must be drawn appropriately based on the data presented.  Reviewer-2’s comment: Partly | Thank you so much for your feedback. We have utilized deductive research approach as this method is suitable for empirical study (please refer to first para of “section 4. Materials and Method”, page 10). Our sample size is 432 which is statistically deemed as acceptable sample size for quantitatve study (Burns et al., 1995). We have described our development of survey instrument is reported in the 2^nd^ paragraph in page 11. We have also reported convergent and discriminant validity to verify construct reliability and validity (page 13-15).  Burns, A. C., Burns, K. R., & Bush, R. F. (1995, March). The SPSS Student Assistant: The Integration of a Statistical Analysis Program into a Marketing Research Textbook. *In Developments in Business Simulation and Experiential Learning: Proceedings of the Annual ABSEL conference (Vol. 22)*. |
| 3. | Has the statistical analysis been performed appropriately and rigorously?  Reviewer-2’s comment: Yes | Thank you so much for your positive compliment. |
| 4. | Have the authors made all data underlying the findings in their manuscript fully available?  Reviewer-2’s comment: No | Thank you for your comment. Relevant data sets (1. Raw data file_csv file, 2. Measurement model_xlsx file, 3. Structural model__xlsx file) have been uploaded in a zip file in the submission platform with the revised manuscript. |
| 5. | Is the manuscript presented in an intelligible fashion and written in standard English?  Reviewer-2’s comment: No | This time, we have taken professional proof read service from a native English scholar to improve the English quality of our revised manuscript (please refer to the yellow mark across the manuscript that reflects the English proof read. |
| 6. | Please use the space provided to explain your answers to the questions above. You may also include additional comments for the author, including concerns about dual publication, research ethics, or publication ethics. (Please upload your review as an attachment if it exceeds 20,000 characters)  Reviewer-2’s comment: There are some grammatical problems in this paper, it should be edited first before submit for our review. For e.g. : The dearth of credible information might lead to form negative impressions among customers. Also, credible source of information also affects users’ perceived ease of use in regard to adopt a new information system.  Quite an ordinary paper, didn't see any contribution from this work. And many others. There are also some redundance sentences. All these problems shown that the writer did not do the work rigorously.  I have no problem if Plos One want to accept this paper but personally I think it needs to be revise thoroughly before it is published. My opinion is either reject or revise thoroughly before submit for review. | Thank you for your comment. We have further taken the English proof-reading service from one academic scholar who is a born English native speaker to improve the English language of our manuscript.  We have carefully edited the entire manuscript. The indicated sentence is now written as “*The lack of credible information might result in negative customer perception*” on page 7 (yellow mark).  Also, the second indicated sentence is now written as “*consumers’ perceptions about perceived ease of use of new information systems are influenced by reliable sources of information*” on pages 7-8 (yellow mark).  The novelty of our research is reported in the second paragraph on page 3. |

**NOTE:** For This study was supported by the School of Management, Jiujiang University, China. There is no grant number issued by the department. In regard to data availability statement, we’ve provided all necessary data files with the manuscript (please refer to 1. Raw data file_csv file, 2. Measurement model_xlsx file, 3. Structural model__xlsx file).

Kind Regards,

The Corresponding Author:

*Ahmed Al Asheq*

1) PhD Student

School of Business

Maynooth University, Ireland.

Email: ahmed.asheq.2022@mumail.ie

2) Lecturer

Department of Business Administration

World University of Bangladesh (WUB), Bangladesh.

Email: aasheq@business.wub.edu.bd

**Other Authors:**

Dr. Md. Atikur Rahaman (email: atik@jju.edu.cn)

H. M. Kamrul Hassan (email: kamrul@cu.ac.bd)

K. M. Anwarul Islam (email: anwarul@themillenniumuniversity.edu.bd)
