# Supplementary material for: The interplay between eWOM information and purchase intention on social media: Through the lens of IAM and TAM theory
Source: PLoS One. 2022 Sep 6;17(9):e0272926. doi: 10.1371/journal.pone.0272926 (PMC9447927; doi:10.1371/journal.pone.0272926)
Supplement: Attachment — Submitted filename: revised Manuscript_PONE-D-21-35683R2_without any mark-up 150722 WLS.docx [file pone.0272926.s005.docx]

**The Interplay between eWOM Information and Purchase Intention on Social Media: Through the Lens of IAM and TAM Theory**

Md. Atikur Rahaman, PhD**^1^**

Associate Professor

School of Management

Jiujiang University, Jiangxi, China. Email: atik@jju.edu.cn

H. M. Kamrul Hassan**^2^**

Associate Professor

Department of Marketing, Faculty of Business Administration

University of Chittagong, Chattogram-4331, Bangladesh.

Email: kamrul@cu.ac.bd

Ahmed Al Asheq**^3**^**

1) PhD Student

School of Business

Maynooth University, Ireland.

Email: ahmed.asheq.2022@mumail.ie

2) Lecturer

Department of Business Administration

World University of Bangladesh (WUB), Bangladesh.

Email: aasheq@business.wub.edu.bd

K. M. Anwarul Islam^4^

Associate Professor

Department of Businesses Administration

The Millennium University

Bangladesh

E-mail: anwarul@themillenniumuniversity.edu.bd

**Corresponding Author: Ahmed Al Asheq

**Abstract:** The maturity and growth of social media have empowered online customers to generate electronic word of mouth (eWOM), on various online websites and platforms, which may influence an individual’s decision-making process. This paper delves into exploring eWOM information’s impact on social media users’ purchase intention by applying the information adoption model (IAM) and the technology acceptance model (TAM). PLS-SEM (SmartPLS V.3.3) has been utilized to test the hypotheses using data of 432 respondents. The research findings evinced that eWOM information quality, eWOM information credibility, usefulness, and ease of use of eWOM information have been critical in determining online consumers’ intention to adopt eWOM and form purchase behavior on social media. The study’s outcomes will offer the marketing managers a viewpoint to realize the significance of the effect of eWOM information on online purchase intention among social media users. Furthermore, the study findings will also enlighten marketing and business managers to utilize social media websites by gauging consumer behavior and focusing on characteristics of eWOM information on social media for better consumer insights.

**Keywords:** eWOM information quality, eWOM information credibility, eWOM information adoption, purchase intention, social media.

**1. Introduction**

The swift development of technological progress has offered customers several modes of communication channels to interact with business firms in today’s time (Ngarmwongnoi, Oliveira, AbedRabbo, & Mousavi, 2020). Along with this technological advancement, various social media platforms have emerged to exchange information among online customers. Globally, social media is considered one of the most powerful platforms among consumers to share and spread information (Bilal, Jianqiu, Dukhaykh, Fan, & Trunk, 2021). The maturity and growth of social media have also empowered online customers to generate electronic word of mouth, known as “eWOM,” on various online websites and platforms, utilizing product/service reviews, blogs, recommendations, and so on (Liu, Ozanne, & Mattila, 2018). Hennig-Thurau, Gwinner, Walsh, and Gremler (2004) defined the concept of “eWOM” as “any positive or negative statement made by potential, actual or former customer which is available to a multitude of people via the internet” (p. 39). The eWOM information is more likely to be reliable as individuals can experience a neutral opinion about a product or service (Doh & Hwang, 2009). Therefore, eWOM information has been discerned as a more powerful medium to influence an individual’s decision-making process (Tien, Rivas, & Liao, 2019). Before purchasing a product or service, consumers tend to look for more information about the product online and significantly consider the review comments about the product generated by other consumers (Jiang et al., 2021). Consumers rely on eWOM information to minimize their doubts and risk when making a purchase effort (Wang, Cunningham, & Eastin, 2015). Past research exhibited that eWOM impacted the consumers’ selection process of goods/services (See-To & Ho, 2014) and influenced individuals’ purchasing decisions (Erkan & Evans, 2016).

Nonetheless, social media websites are considered a relatively new platform for generating eWOM, allowing online users to develop a connection with other users by sharing their opinions, ideas, and experiences (Erkan & Evans, 2018). Through social media engagement, people can now exchange their viewpoints and share experiences about a product/service with their friends and familiar people in the network (Zha, Yang, Yan, Liu, & Huang, 2018). This reciprocal information-sharing process in social media makes eWOM information more authentic and dependable for the consumers. The social media-generated conversations were found to have a robust natural impact on online purchase behavior (Roy, Datta, & Mukherjee, 2019). As online individuals form their purchase decisions based on eWOM information, it is also critical for them to sort out and screen out the relevant information available online before using as online users are generally exposed to a boundless quantity of eWOM information on social media websites. Still, the aspects of eWOM information and online purchase intention at social media websites are not well explored in the literature. More specifically, the roles of eWOM information in triggering consumer behaviors such as buying intention have not been largely investigated in regard to the eWOM perspective (Leong, Loi & Woon, 2021; Anubha, 2021; Cheung & Thadani, 2012). Furthermore, Knoll (2016) argued that the influence of eWOM not only relies on the information but also depends on online users on social media. Hence, it is of importance to evaluate the influence of various aspects of eWOM information on consumer purchase intention from the social media perspective. To achieve this research aim, both Information Adoption Model (IAM) and Technology Acceptance Model (TAM) have been considered as it is believed that IAM is an appropriate model to illustrate the aspects of eWOM information. In contrast, TAM will explain online consumer behavior towards eWOM.

The current paper presents a number of valuable and noteworthy contributions to the current literature based on the consumer behavior perspective. Firstly, several studies have examined the impact of eWOM on consumer purchase intention by considering the TAM model (Sardar, Manzoor, Shaikh & Ali, 2021; Song, Liew, Sia & Gopal, 2021), in which factor: “perceived usefulness” of the TAM model was adopted as a determinant of information adoption and purchase intention. Still, the impact of perceived ease of use of eWOM on consumer purchase decisions was merely reported in the literature. The novelty of this study lies in attempting to fill out this gap by analyzing the influence of ease of use of eWOM on information adoption behavior and purchase intention. Secondly, this study has considered a recent growing economy: Bangladesh, to draw the study sample as it is observed that the country has been experiencing a digital transformation across the nation (Humida, Al Mamun & Keikhosrokiani, 2021), where eWOM plays a critical role in developing the business growth, especially on online platforms. Lastly, several studies in Bangladesh were carried out to exhibit the connection of social media with brand equity (Hafez, 2021), consumer behavior (Eti, Horaira & Bari, 2021), and purchase intention (Sarker & Khayer, 2021; Al Amin, Nowsin, Hossain & Bala, 2020). In contrast, the relationship between various attributes of eWOM and consumer behavioral aspects was rarely tested in the Bangladesh context, where social media-based businesses have started flourishing day by day. Henceforth, the current study has filled this void by integrating the IAM and TAM models to predict consumer purchase intention In Bangladesh. The findings of this paper also offer theoretical knowledge on the roles of eWOM information on consumer behavior in social media and strive to contribute to the body of literature through the research model. Finally, as a means of managerial implications, examining the impact of aspects of eWOM information on purchase intention in social media context would offer online consumer insights to the marketing managers to develop a better marketing strategy by leveraging eWOM information.

2. Electronic Word of Mouth (eWOM) in Social Media

The eWOM information can be referred as any form of comment regarding a product or service, which is easily accessible and available to numerous individuals on internet-mediated platforms (Gvili & Levy, 2018). Social media websites have offered a new platform for the communication channel to disseminate information, and these websites are regarded as one of the suitable and appropriate platforms for electronic word of mouth (eWOM) (Herrero & Martínez, 2021). On these websites, individual users can easily make any comments and share any information in the form of text writing, posting a photo or uploading a video, etc. Apparently, enriched contents on social media websites result in making eWOM information more noticeable and entertaining to online users (Zhou, Yan, Yan, & Shen, 2020). On social media websites, online users usually like to read and check various comments, thoughts, and others’ experiential opinions about products or services (Bilal et al., 2021). Therefore, online consumers are more intentional to resort to diverse platforms of social media in order to gather the needful information about brands (Barreda, Bilgihan, Nusair, & Okumus, 2015).

Generally, eWOM information may emerge in several means on social media platforms, such as online users can deliberately share their opinions about brands by posting on social media, or they can reveal their liking for any brand by becoming a member of an online fan club. Even marketing people can purposefully share various helpful information about the brands on the official page of their social media accounts (Alboqami et al., 2015). So, eWOM information might have an important influence on online consumers to decide which products they will purchase. As eWOM helps individuals to make better purchase decision (Bronner & De Hoog, 2011), hence it is very critical to find out what aspects or characteristics of eWOM information might affect consumers’ purchase intention on social media websites.

3. Literature Review & Hypotheses Development

3.1. Theoretical Underpinning

Several research studies have adopted the information adoption method to evaluate how individuals make use of the information they receive or the message they convey to themselves (Nonaka, 1994). Many researchers in the discipline of information systems have adopted the concept of the “Information Adoption Model (IAM)” to demonstrate how humans receive persuasive information in order to make decisions (Watts & Wyner, 2011; Zhu, Chang, & Luo, 2016). Sussman and Siegal (2003) proposed the theory of IAM, which was originally formulated on the basis of the integration of both the “Elaboration Likelihood Model (ELM)” (Petty & Cacioppo, 1986) and the “Technology Acceptance Model (TAM)” (Davis, 1989). This IAM model demonstrates that individuals are significantly influenced by a set of information in two different ways: either through the central path or through the peripheral path (Shen, Cheung, & Lee, 2013). The core of the communication is shown by the central path, whereas the peripheral path is not directly related to the central part of the message (Shu & Scott, 2014). The IAM is often combined of four attributes, which are argument quality (which corresponds to the central route), source credibility (which corresponds to the peripheral pathway), functionality and incorporation of the information (which refers to the periphery path), and overall effectiveness (Khwaja, Mahmood, & Jusoh, 2020). IAM aims to demonstrate how people tend to be impacted by computer-mediated information on internet-based platforms by addressing these four factors (Erkan & Evans, 2016). Thus, the IAM model notably determines the trustworthiness and quality of eWOM information.

Alternatively, the “Technology Acceptance Model (TAM)” developed by Davis (1989), is one of the most commonly used theories that truly describes any behavioural attributes of information technology (IT) users when they encounter with the task of embracing and acknowledging a modern IT-oriented platform (Kamal, Shafiq, & Kakria, 2020; Lee, Kim, & Hackney, 2011). Despite the fact that this model was originally formed from the “Theory of Reasoned Action (TRA),” TAM theory has been continued to be based on information technology (Park & Kim, 2014), in contrast to the way that TRA theory has tended to be restricted to behavioral models (Zkan et al., 2010). It is generally built on two constructs: perceived usefulness and perceived ease of use, both of which are utilized to forecast an individual’s likelihood to embrace a technology system (Tarhini, Arachchilage, & Abbasi, 2015). There has been a substantial application of this approach in various settings, including social media usage (Alenazy, Al-Rahmi, and Khan, 2019), mobile banking usage (Kumar, Lall & Mane, 2017), and online review (Elwalda, Lü, & Ali, 2016). This study examines the usefulness and ease of use of eWOM information obtained via TAM to determine whether it impacts online purchase intention.

3.2. Hypotheses Development

3.2.1. The Relationship of eWOM Information Quality (IQ) with Perceived Usefulness (USE) and Ease of Use of eWOM Information (EOU)

Information quality (IQ) indicates the users’ perception-based assessment of whether the given information attributes fulfill their usage purpose or meet up the needs of any given system (Jiang et al., 2021). Information quality (IQ) represents the correctness, lucidity, understandability, and dependability of the given information embedded into a system (Chen & Chang, 2018). Dancer, Filieri and Grundy (2014) described that consistency and completeness had been considered the two critical determining factors of information quality. According to Savolainen (2011), “The issues of information quality and credibility are gaining importance, particularly in the World Wide Web context. The WWW provides unique information-seeking environment but often lacks quality control mechanisms” (p. 1243). IQ plays a crucial part in assessing and appraising the quality of the products or services by the customers on an online platform (Filieri & McLeay, 2014). When high-quality information is delivered to consumers through social media platforms, they are more likely to perceive it as valuable and useable, encouraging them to make a more informed purchase decision. The excellent review comments and conversational postings on a particular subject assist consumers in locating useful information and give some critical suggestions on the subject (Zheng, Zhao, & Stylianou, 2013). Furthermore, once internet users come across improved quality information, they consider the content convenient and valuable (Kim, Chung, Lee, & Preis, 2016). By adopting the UTAUT model, Lee, Sung, and Jeon (2019) found that IQ positively affects the usefulness and ease of use of the given information in the food delivery service application. Henceforth, based on the discussion, the study assumes that the IQ of eWOM information will determine both perceived usefulness and ease of use of eWOM information from the social media purchasing perspective. Hence, the following assumptions are proposed:

*H1: eWOM Information quality (IQ) will affect the perceived usefulness of eWOM information (USE).*

*H2: eWOM Information quality (IQ) will affect the perceived ease of use of eWOM information (EOU).*

3.2.2. The Relationship of eWOM Information Credibility with Usefulness and Ease of Use of eWOM information

In essence, credibility may be defined as the capacity to be believed from the users’ perspective (Yin, Sun, Fang & Lim, 2018). Information credibility can be explained as the consumers’ perception and discernment of the genuineness and trustworthiness of the given information on an electronic platform such as social media (Jiang et al., 2021). Generally, information credibility indicates the extent to which a person considers the given information believable and convincing provided by the respective electronic source (Chen, Tao, & Zhou, 2019). The eWOM information credibility measures to what extent an online user considers the review comments or recommendations from other online users as genuine and factual (Cheng & Zhou, 2010). Consumers usually tend to prioritize information that they feel is more believable and credible when it is available on an online platform (Verhellen, Dens & De Pelsmacker, 2013), and internet users are more expected to perceive authentic information as more useful and credible (Song, Zhang, & Yu, 2020). The lack of credible information might result in negative customer perceptions (Hajli, 2018). Sweeney, Soutar and Mazzarol (2012) argued that the acceptance of review comments by individuals also relies on their value judgment of the credibility of the provided information in a message. Matute, Polo-Redondo and Utrillas (2016) advocated that eWOM has appeared as a significant medium of customer information in online purchasing. On social media platforms, online users share their viewpoints, opinions, judgments, and their negative or positive perspectives toward a particular product. This type of opinion-based eWOM information enhances the online users’ perceived usefulness as they believe that the information is unbiased and credible (Yeap, Ignatius, & Ramayah, 2014). As a result, consumers’ perceptions about perceived ease of use of new information systems are influenced by reliable sources of information (Li, 2013). Therefore, the study proposes the following hypotheses:

*H3: Information credibility (IC) will affect the perceived usefulness of eWOM information (USE).*

*H4: Information credibility (IC) will affect the perceived ease of use of eWOM information (EOU).*

3.2.3. The Relationship of Perceived Ease of Use of eWOM Information (EOU) with the adoption of eWOM information (INAD) and Purchase Intention (PI) on Social Media

Davis (1989) interpreted the idea of “perceived ease of use” as “the degree to which a person feels that utilizing a given system would be devoid of effort” (p. 320). From a consumer point of view, perceived ease of use assesses how effortless it is to utilize a specific technology-based system (Hajiheydari & Ashkani, 2018). In the current paper, perceived ease of use of the information relates to online consumers’ view that the online information may be readily and seamlessly comprehended and utilized to increase the consumer’s performance in the online purchase decision-making process. Many researchers have indicated that perceived ease of use significantly impacts behavioral adoption and intention. For example, in his research study, Qashou (2021) demonstrated that perceived ease of use positively impacted students’ attitudes toward adopting mobile learning apps. Al-Emran and Teo (2020) empirically analyzed Omani students’ acceptance of e-learning systems. They discovered that simplicity of use significantly impacts students’ readiness to embrace e-learning systems. According to the study findings conducted by Bonn, Kim, Kang and Cho (2016), ease of use is a determinant of online intention to purchase wine among online-based customers. Thus, based on the existing literature, it is believed that if eWOM information becomes easier and uncomplicated for online users on social media networks, online customers will be more likely to adopt eWOM information and will have an intention towards purchase the products or services on social media websites. Henceforth, the current paper postulates the following hypotheses:

*H5: Perceived ease of use of eWOM information (EOU) will affect eWOM information adoption (INAD).*

*H6: Perceived ease of use of eWOM information (EOU) will affect purchase intention(PI) on social media.*

3.2.4. The Relationship of Perceived Usefulness of Information (USE) with Adoption of eWOM Information (INAD) and Purchase Intention (PI) on Social Media

As defined by Davis (1989), “perceived usefulness” is “the degree to which a person feels that utilizing a certain system would improve his or her job performance” (p. 320). According to de Luna, Liébana-Cabanillas, Sanchez-Fernández and Munoz-Leiva (2019), Perceived Usefulness has been defined as the user’s belief that using online technology will lead to achieving their performance. According to Cheung, Lee, and Rabjohn (2008), the perceived usefulness of the information indicates the users’ perception that new information is likely to upgrade their performance. In the case of online purchases, information adoption is driven by the degree to which the provided information is valuable on the specific online platform (Abedi, Ghorbanzadeh, & Rahehagh, 2019). If online consumers consider the information, they find on social media networks to be relevant and meaningful, they are more likely to have formed a solid inclination to accept that knowledge (Erkan & Evans, 2016). Furthermore, prior research identified perceived usefulness as a significant predictor of online purchase intention (Kucukusta, Law, Besbes & Legohérel, 2015; Moslehpour, Pham, Wong & Bilgiçli, 2018; Ventre & Kolbe, 2020). Therefore, the study proposes the following hypotheses:

*H7: Perceived usefulness of eWOM information (USE) will affect eWOM information adoption (INAD).*

*H8: Perceived usefulness of eWOM information (USE) will affect purchase intention (PI) on social media.*

Based on the above hypotheses, the following figure is proposed as the conceptual model of the study (please refer to figure 1):

*H7*

*H5*

*H8*

*H6*

*H9*

*H1*

*H2*

*H4*

*H3*

Figure 1: Conceptual Model of the Study

Why here??? Should be after H9

3.2.5. The Relationship of Adoption of eWOM Information (INAD) with Purchase Intention (PI) in Social Media

The Information adoption can be referred to as a process through which individuals are purposefully involved in making use of the given information (Cheung et al., 2008). Information adoption has been regarded as one of the critical factors in impacting consumers’ intention towards making a purchasing decision (Cheung & Thadani, 2012). The adoption of eWOM information is based on the information adoption procedure that indicates the degree of the online users’ utilization process of given information (Daowd et al., 2020). It is highly likely that individuals who adopt eWOM information tend to leverage it when making an actual buying decision (Daowd et al., 2020). Online users, who are likely to utilize and adopt eWOM information, tend to have buying intentions (Erkan & Evans, 2018). Consumers will develop a firm purchase intention if they get involved in eWOM information (Yusuf & Busalim, 2018). Several studies reported a positive connection between eWOM and purchase intention in the online platform (Choi, 2021; Liao, Hu, Chung, & Huang, 2021; Rimadias, Herlambang, Muladi, & Dharma, 2021). Therefore, based on the discussion on the connection between eWOM information and purchase intention, the following hypothesis is postulated:

*H9: The adoption of eWOM information (INAD) will affect consumers’ purchase intention (PI) on Social Media.*

4. Materials and Methods

From the research philosophy perspective, the positivism paradigm has been considered by the researcher to focus on facts with quantitative analysis in order to operationalize concepts so that they can be measured scientifically and systematically (Gray, 2013). Therefore, it is essential to incorporate deductive methods emphasizing truly quantitative analysis and then test by empirical observation (Collis & Hussey, 2013) to achieve predetermined well-defined research objectives.

4.1. Study Population & Sample

For the current investigation, university students studying in various public and private universities inside Chattogram city of Bangladesh have been considered the sampling population in this study. University students represent a diverse group of different qualities and reflect the broader community and society (Hanzaee & Esmaeilpour, 2017). The convenience sampling method has been considered for this study as it allows the researcher to attain specific data and patterns without the inconvenience of a randomized sample. Additionally, this sampling method assists in capturing the presence of a certain quality of behavior within the given sample, along with identifying associations between various phenomena (Etikan, Musa & Alkassim, 2016). An online survey was conducted around May and June 2021 to obtain the required data. After eliminating 118 incomplete responses from a total of 550 questionnaires, 432 sample respondents have been chosen, resulting in an effective sample rate of about 78.55 percent. Participants were initially approached through a direct email to all university students notifying them that they had been chosen randomly to participate in this survey. Later they received an email including a link to Google form, where a brief explanation of the study was given first, and then asked to complete a questionnaire survey with demographic questions. The email also contained the researcher’s contact information that students can use to reach the researcher. The online survey was highly compatible with widely used web browsers, and the surveys were easily understandable using a large font.

4.2. Measures

A survey instrument was developed based on earlier research which included relevant information related to social media usage and verified in two stages. Initially, the questionnaire set was delivered to researchers and professionals in the business and IT industries who have expert knowledge on such topics to ensure its simplicity and relativeness with the field. The experts’ recommendations and opinions have been utilized to improve the questionnaire. Later, pilot testing was performed on a small sample of 35 respondents having university degrees and using social media-related applications, who were asked for their thoughts to minimize the complexity and ambiguity of the questionnaire. SPSS-23, together with Cronbach’s alpha, was used to calculate reliability. All the variables are evaluated without any change as they all surpassed the threshold value of the Cronbach alpha reliability (0.70). In the final analysis, the data from the pilot research were not used.

In this study, the questionnaires consisted of two distinct parts: part A is about the demographic profile of the participants. At the same time, section B comprised dependent and independent variables derived from various previous studies. For measuring constructs of eWOM information quality, three items were adopted from Park, Lee and Han (2007); four items are considered for measuring eWOM information credibility, which is adopted from Prendergast, Ko and Siu Yin (2010); three items are considered for measuring eWOM information usefulness, which is adopted from Sussman and Siegal (2003); two items are considered for measuring eWOM information ease of use, which is adopted from Davis (1989). Four items have been adopted for the measurement of eWOM information adoption (Fang, 2014). Finally, four items were adopted from Coyle and Thorson (2001) to measure the purchase intention on social media websites. Participants were asked to rate the items prepared based on the five-point rating Likert scale (starting with strongly disagree to strongly agree).

4.3. Data Analysis

Structural equation modeling is a well-developed scientific approach that truly emphasizes statistical tools to develop cause and effect relationships between independent and dependent constructs used for the measurement of study through developing predetermined critical hypotheses logically and analytically (Bagozzi & Yi, 2012; Musil, Jones, & Warner, 1998). This study has employed SPSS 23 for descriptive analysis, SMARTPLS 3.3 for confirmatory factor analysis, and a structural model.

5. Results

5.1. Respondents’ Profile

**Table-1:** Demographic Information

| Variables | Items | Frequency | Percent |
| --- | --- | --- | --- |
| Age | Below 20 years | 69 | 15.97% |
|  | 20 - 25 years | 144 | 33.33% |
|  | 25 - 30 years | 182 | 42.13% |
|  | Above 30 years | 37 | 8.56% |
| Gender | Female | 165 | 38.19% |
|  | Male | 267 | 61.81% |
| Education Level | Undergraduate | 187 | 43.29% |
|  | Postgraduate | 245 | 56.71% |
| Social media using experience | Less than 1 year | 132 | 30.56% |
|  | 1–3 years | 273 | 63.19% |
|  | >3 years | 27 | 6.25% |

**n=432

The demographic profile of respondents has been taken into consideration based on gender, age, level of education, and social media user experience (table 1). Overall, the survey results showed that 61.81% of the participants were males, 42.13% were between the ages of 25 and 30, 56% were studying at the postgraduate level, and 63.19% had 1-3 years of usage experience of social media.

Confirmatory Factor analysis has been employed to evaluate the validity and reliability of overall and individual measurement constructs. In CFA, a predetermined hypothesis is estimated to lead to a certain outcome based on an underlying causal explanation. In order to evaluate the measurement model, outer loading constructs reliability and construct validity were examined. To determine the correlation between the latent measures and the reflective indicators, the values of the outer loadings were evaluated where indicators with outer loading of 0.6 or above were considered (Hair, Hult, Ringle, & Sarstedt, 2021). Cronbach’s alpha values were used to determine the construct’s reliability, where all values demonstrated satisfactory results, exceeding the 0.60 criterion (Hair et al., 2021). The current study has employed convergent and discriminant validity to verify construct validity. While evaluating convergent validity, values of AVEs were between 0.565 and 0.753, and the CR values are also found within 0.796 to 0.869, which correlates to the threshold value explained by Hair et al. (2021) (see table 2).

5.2. Measurement Model Analysis

**Table 2:** Construct Validity Results

| Constructs | Items | Loadings | Cronbach’s Alpha | CR | AVE |
| --- | --- | --- | --- | --- | --- |
| eWOM Information quality (IQ) | IQ1 | 0.82 | 0.774 | 0.869 | 0.688 |
|  | IQ3 | 0.858 |  |  |  |
|  | IQ4 | 0.809 |  |  |  |
| eWOM Information credibility (IC) | IC1 | 0.774 | 0.77 | 0.853 | 0.592 |
|  | IC2 | 0.757 |  |  |  |
|  | IC3 | 0.823 |  |  |  |
|  | IC4 | 0.72 |  |  |  |
| eWOM Ease of use (EOU) | EOU1 | 0.844 | 0.675 | 0.859 | 0.753 |
|  | EOU2 | 0.891 |  |  |  |
| eWOM Usefulness (USE) | USE1 | 0.759 | 0.615 | 0.796 | 0.565 |
|  | USE2 | 0.776 |  |  |  |
|  | USE3 | 0.72 |  |  |  |
| eWOM Information Adoption (INAD) | INAD1 | 0.747 | 0.78 | 0.858 | 0.603 |
|  | INAD2 | 0.807 |  |  |  |
|  | INAD3 | 0.827 |  |  |  |
|  | INAD4 | 0.72 |  |  |  |
| Purchase Intention (PI) | PI1 | 0.7 | 0.793 | 0.866 | 0.619 |
|  | PI2 | 0.831 |  |  |  |
|  | PI3 | 0.823 |  |  |  |
|  | PI4 | 0.787 |  |  |  |
|  |  |  |  |  |  |

After concluding the convergent validity test, the discriminant validity test was performed. As shown in the literature, Fornell and Larcker (1981) stated that for each indicator, the square root of AVE must be higher than the associated values of that specific indicator with other indicators (Table 3). Despite this, many argue that the Fornell–Larcker criteria are not justifiable since they cannot correctly evaluate the presence of discriminant validity in extensive research (Henseler, Ringle, & Sarstedt, 2015). To address the shortcomings of the Fornell and Larcker (1981) method, Henseler et al. (2015) used the HTMT as a more thorough and more confined approach for researchers employing PLS-SEM to evaluate discriminant validity, specifying that the HTMT values must be less than 0.85 or 0.90 (Henseler et al., 2015). The HTMT test result is presented in Table 3, and the results met the HTMT 0.85 and HTMT 0.90 criteria, indicating that the measurement model was discriminately validated.

**Table-3:** Discriminant validity

| Fornell and Larcker’s criterion | | | | | | | | | |  |  |
| --- | --- | --- | --- | --- | --- | --- | --- | --- | --- | --- | --- |
|  | IQ | IC | | EOU | | | USE | INAD | | PI | |
| IQ | 0.829 |  | |  | | |  |  | |  | |
| IC | 0.371 | 0.769 | |  | | |  |  | |  | |
| EOU | 0.395 | 0.557 | | 0.868 | | |  |  | |  | |
| USE | 0.33 | 0.416 | | 0.405 | | | 0.752 |  | |  | |
| INAD | 0.52 | 0.451 | | 0.552 | | | 0.489 | 0.777 | |  | |
| PI | 0.334 | 0.375 | | 0.457 | | | 0.517 | 0.492 | | 0.787 | |
| Heterotrait-Monotrait Ratio (HTMT) | | | | | | |  |  |  | |  |
|  | | | IQ | | IC | EOU | USE | INAD | | PI | |
| IQ | | |  | |  |  |  |  | |  | |
| IC | | | 0.476 | |  |  |  |  | |  | |
| EOU | | | 0.535 | | 0.765 |  |  |  | |  | |
| USE | | | 0.482 | | 0.595 | 0.624 |  |  | |  | |
| INAD | | | 0.667 | | 0.575 | 0.752 | 0.701 |  | |  | |
| PI | | | 0.42 | | 0.473 | 0.612 | 0.734 | 0.614 | |  | |

5.3. Structural Model Analysis

An assessment of the structural model was undertaken to determine the significance of the paths and the predictive power of the model through the PLS algorithm, and then by considering a bootstrapping using 5,000 samples process that involved random resamples from the original data set to determine the significant levels of path coefficients (Hair Jr et al., 2021) (Figure 2). According to the findings (table 4), eWOM information quality significantly influences both eWOM perceived ease of use and eWOM perceived usefulness (β = 0.219, t-values =4.882, significance p < 0.000 and β = 0.204, t-values =3.996, significance p < 0.000), therefore confirming H1 and H2. Again, perceived ease of use and perceived usefulness of eWOM information were influenced by eWOM information credibility (β = 0.476, t-values= 6.691, significance p < 0.000 and β = 0.341, t-values= 5.396, significance p < 0.000), thereby validating H3 and H4. Ease of use of eWOM has shown significant influence on both eWOM information adoption and purchase intention (β =0.424, t-values=8.514, significance p < 0.000 and β =0.203, t-values=3.703, significance p < 0.000), and confirmed the H5 and H6.

**Figure 2**: Path Coefficients of the Model

Again, eWOM perceived usefulness had positive and significant influence on both eWOM information adoption and purchase intention (β =0.317, t-values= 5.913, significance p < 0.000 and β =0.327, t-values =6.196, significance p < 0.000), and confirmed the H7 and H8. Finally, H9 is accepted as eWOM information adoption and purchase intention are found significant (β = 0.221, t-values= 3.227, significance p < 0.05).

| **Table-4:** Path Coefficients | | | | | | |
| --- | --- | --- | --- | --- | --- | --- |
| Hypothesis | Path | Beta | SE | T-value | P Values | Decision |
| H1 | IQ -> EOU | 0.219 | 0.045 | 4.882 | 0.000 | Accepted |
| H2 | IQ -> USE | 0.204 | 0.051 | 3.996 | 0.000 | Accepted |
| H3 | IC -> EOU | 0.476 | 0.057 | 8.388 | 0.000 | Accepted |
| H4 | IC -> USE | 0.341 | 0.063 | 5.396 | 0.000 | Accepted |
| H5 | EOU -> INAD | 0.424 | 0.050 | 8.514 | 0.000 | Accepted |
| H6 | EOU -> PI | 0.203 | 0.055 | 3.703 | 0.000 | Accepted |
| H7 | USE -> INAD | 0.317 | 0.054 | 5.913 | 0.000 | Accepted |
| H8 | USE -> PI | 0.327 | 0.053 | 6.196 | 0.000 | Accepted |
| H9 | INAD -> PI | 0.221 | 0.068 | 3.227 | 0.001 | Accepted |

The determination coefficient (R^2^ value) is the most broadly utilized measure to analyze the structural equation model. The R^2^ value measures the goodness of the structural model. The R^2^ value of 0.408 means that the model explains that 37.0 percent of the variance in young consumers’ behavior intention towards social media can be explained by the predictors in this study. Furthermore, the study assessed effect sizes (f^2^) and predictive relevance by employing the Stone- Geisser Q^2^ value. The “ƒ²” effect size is utilized to measure the degree to which each latent exogenous variable influences the latent endogenous variables, where the satisfactory effect size values are 0.35, 0.15, and 0.02 deemed as substantial, medium, and small effect sizes, respectively (Hair Jr et al., 2021). Again, Hair Jr et al. (2021) suggested that predictive relevance Q^2^ values should be greater than 0, which means that the exogenous variables have predictive significance over the endogenous variables and the model is predictively relevant. Table 5 summarizes the acceptable values for predictive relevance Q^2^, effect sizes (f^2^), and coefficient of determination R^2^.

**Table-5:** predictive relevance *Q^2^*, effect sizes (*f^2^*), and coefficient of determination *R^2^*

|  | *R^2^* | *Q^2^* | *f^2^* | Decision |
| --- | --- | --- | --- | --- |
| Purchase Intention | 0.37 | 0.219 |  |  |
| eWOM Information Adoption |  |  | 0.047 | Small |
|  |  |  |  |  |
| eWOM Information Adoption | 0.389 | 0.227 |  |  |
| Perceived Ease of Use of Information |  |  | 0.246 | Medium |
| Perceived Usefulness of Information |  |  | 0.138 | Small |
|  |  |  |  |  |
| Perceived Ease of Use of Information | 0.351 | 0.26 |  |  |
| Information Quality of Information |  |  | 0.064 | Small |
| Information Credibility of Information |  |  | 0.301 | Medium |
|  |  |  |  |  |
| Perceived Usefulness of Information | 0.209 | 0.116 |  |  |
| Information Quality of Information |  |  | 0.045 | Small |
| Information Credibility of Information |  |  | 0.127 | Small |

6. Discussion & Implications

This study aims to examine the behavioral intention to order in social media based on the eWOM review through integrating the “Information Adoption Model (IAM)” and “Technology Acceptance Model (TAM)”. Based on this, the current framework incorporates eWOM information quality, eWOM information credibility, perceived ease of use of information, perceived usefulness of information, adoption of eWOM information, and purchase intention in social media. Incorporating eWOM information quality and eWOM information credibility as a context creates an extended framework of TAM, which has enhanced the model’s predictive ability (*R^2^* = 37%).

The eWOM information quality significantly affects information usefulness and ease of use which is relevant to the previous studies (Kang & Namkung, 2019; Riana, Hidayanto, Hadianti, & Napitupulu, 2021; Spatar, Kok, Basoglu, & Daim, 2019). The eWOM information quality significantly affects information usefulness and ease of use which is relevant to the previous studies (Kang & Namkung, 2019; Riana et al., 2021; Spatar et al., 2019). With regard to the online-based platform, users are more willing to take advantage of information quality if they believe that the system delivers clear, comprehensible, and current information and the content is of high quality (Roca, Chiu, & Martínez, 2006). The results from the structural equation model also indicated that the eWOM information credibility significantly affects information usefulness and ease of use which is significant to earlier studies ((Filieri, Acikgoz, Ndou & Dwivedi, 2020). The credibility of the provided information is crucial for improving the benefit of a delivery channel when information overflow makes selection challenging for consumers, and consumers feel that if an online platform is convenient and efficient, it has the ability to manage successful transactions (Kang & Namkung, 2019). Information on social media is primarily commercialized, making verifying information’s quality, traceability, and credibility difficult. Therefore, reviews on social media platforms greatly assist online users in determining the traceability, authenticity, and quality of online businesses, which in turn encourage them to make a decision. In addition to the reviews provided by consumers, the firm’s engagement in social media communication will help improve its credibility in affecting consumer perceptions (Šerić & Praničević, 2018).

As hypothesized, perceived ease of use had a statistically significant positive effect on behavioral intention to order in social media based on an eWOM review (Cho & Sagynov, 2015; Filieri et al., 2020). The result shows that customers with a positive mindset towards adopting new technologies and who believe that technologies are simple to use may enhance their interest in technology adoption. And if the actual content is simple and fast retrievable, young consumers become more interested in staying on the platform. Thus, they will likely continue adopting it (Filieri et al., 2020). From the outcomes of this study, it has also been revealed that PEU positively influences users’ behavioral intention, which is consistent with previous research studies (Chua, Chiu & Chiu, 2020; Moslehpour et al., 2018). Young consumers will enjoy sharing eWOM on social networks if the social media content is simple and easy to use, combines consumer-focused technology with socialization patterns, and doesn’t limit capabilities for users to post, share, and maintain relationships (Bilal et al., 2021).

Perceived usefulness is significantly related to information adoption, which is significant to previous studies (Hussain, Ahmed, Jafar, Rabnawaz, & Jianzhou, 2017; Shen, Zhang & Zhao, 2016; Zhang, Ito, Wu & Li, 2017). The perceived usefulness of harnessing social media in accomplishing tasks may be interpreted as the adoption of information by users that would help them better complete their tasks (Ayeh, 2015). Information that is useful is considered the critical determining factor influencing individuals to adopt that information (Hussain et al., 2017; Shen et al., 2016). Again, perceived usefulness is significantly associated with behavioral intention (Israel, Zerres & Tscheulin, 2019). In particular, while communicating on the eWOM platform, its usefulness substantially influences people’s perception of using technology and their intention to adopt eWOM (Yang, 2017). Individuals using social networks are introduced to a large volume of eWOM information, which might lead to a higher inclination to incorporate information that seems beneficial to them (Abedi et al., 2019).

Furthermore, it has been noted that information adoption is significantly and positively related to online purchase intention, which supports earlier studies (Abedi et al., 2019; Dang, Wang & Vu, 2020). Information adoption is commonly acknowledged as something that will enable or hinder decisions (Zhao, Wang, Fang & Jin, 2018). The usefulness of information is crucial in purchasing decisions since it aids consumers in determining and understanding products, services, and associated aspects of their purchasing decisions (Shen et al., 2016). When individuals adopt information about the rival brand and the attributes and benefits of a product or service, this enhances their buying decisions since it facilitates them in evaluating their options (Dang et al., 2020). The finding revealed that information adoption partly mediates the suggested relationship (Dang et al., 2020).

In comparison to direct effects, the mediation analysis revealed that the impact of perceived ease of use and perceived usefulness on behavioral intention was mediated by information adoption. Although the contrast between the direct effects of perceived ease of use and perceived usefulness on behavioral intention and the indirect effects through information adoption indicates that the indirect effect was lower, however, the partial mediation and significant relationship reveal that information adoption has a significant role in affecting consumers’ behavioral intention. Overall, the results of the current study point to the importance of eWOM contents in social media, i.e., quality and credibility, as well as to perceived usefulness and ease of use associated with information adoption, which eventually affects behavioral intention toward ordering in social media based on eWOM review.

The current research findings offer the marketing managers a viewpoint to realize the significance of the effect of eWOM information on online purchase intention among social media users. As this research reports that online purchase intention among social media users is connected with eWOM information, hence it does indicate that marketers create brands by leveraging social media platforms. The research findings suggest that both eWOM information credibility and quality enhance the ease of use and usefulness of eWOM information that would affect adoption and purchase intention. So, marketers need to place reliable and quality information about their products or services on social media websites so that it might lead to developing a better impression in online consumers’ mindsets. To enhance the online consumers’ likelihood to purchase, marketers should focus on uploading useful and understandable content to promote their product/services on social media websites to enhance online consumers’ propensity to buy the product. Social media users usually seek more information before making a purchase decision online; thus, business firms may think of developing fan pages to share the latest update about product/service and presenting genuine and complete information to online consumers on social media websites. Furthermore, business managers and marketers should close track and monitor the consumer-generated eWOM on social media to keep themselves updated about consumers’ current viewpoints. To do this, business firms can hire online customer managers, who will assist online consumers by providing helpful, authentic, trustworthy, quality, and intelligible information as per consumer’s requirements so that consumers would provide positive eWOM about the company or product, which will affect other consumers’ purchasing behaviors on social media websites.

7. Conclusions & Directions for Future Research

The current paper has set out the objective to explore the influence of various aspects of eWOM information purchase intention on social media websites by considering relevant components from IAM and TAM models. The study’s research model has been validated through PLS-SEM, where social media using 432 university students participated as respondents. The research findings indicate that quality, credibility, usefulness, and ease of use of eWOM information have been critical in determining online consumers’ intention to adopt eWOM and form purchase behavior on social media. From the managerial perspective, it has been pointed out that marketing and business managers should utilize social media websites to gauge consumer behavior by focusing on characteristics of eWOM information on social media to obtain better consumer insights. Nonetheless, the present research has few limitations. Hence, the research findings of this paper should be considered by taking into account the following research limitations. Firstly, the study developed a research model and measured the model using the PLS-SEM method, which is a quantitative approach. Future studies may consider a qualitative approach to find some new characteristics of eWOM information which may influence online consumer behavior. Secondly, the sample was drawn from social media using university students in Bangladesh, which might not represent the entire population. Thus, considering samples from different age groups in future studies would be more helpful in producing more reflective and generalizable findings. Also, age can be regarded as a moderating variable to capture the differential age effect on the relationship between eWOM information and online consumer behavior. Finally, the study has drawn samples from Bangladeshi university students; hence future studies may adopt samples from developing and developed countries to understand the influence of cultural differences on the relationship between determinants of eWOM information and social media consumer behavior. Also, to generate a greater degree of generalization of the study findings, the current study has not solely focused on a particular product category which might also be considered a limitation of the study. Future studies might consider the specific product category to reveal the impact of eWOM-related characteristics on consumer buying behavior

*References*

Abedi, E., Ghorbanzadeh, D., & Rahehagh, A. (2019). Influence of eWOM information on consumers’ behavioral intentions in mobile social networks: Evidence of Iran. *Journal of Advances in Management Research, 17*(1), 84-109.

Al Amin, M., Nowsin, N., Hossain, I., & Bala, T. (2020). Impact of social media on consumer buying behaviour through online value proposition: A study on e-commerce business in Bangladesh. *BUFT Journal of Business & Economics*, 1, 209–228.

Al-Emran, M., & Teo, T. (2020). Do knowledge acquisition and knowledge sharing really affect e-learning adoption? An empirical study. *Education and information technologies, 25*(3), 1983-1998.

Alboqami, H., Al-Karaghouli, W., Baeshen, Y., Erkan, I., Evans, C., & Ghoneim, A. (2015). Electronic word of mouth in social media: the common characteristics of retweeted and favourited marketer-generated content posted on Twitter. *International Journal of Internet Marketing and Advertising, 9*(4), 338-358.

Alenazy, W. M., Al-Rahmi, W. M., & Khan, M. S. (2019). Validation of TAM model on social media use for collaborative learning to enhance collaborative authoring. *IEEE Access, 7*, 71550-71562.

Anubha (2021). Mediating role of attitude in halal cosmetics purchase intention: an ELM perspective. *Journal of Islamic Marketing*, Vol. ahead-of-print No. ahead-of-print. https://doi.org/10.1108/JIMA-04-2021-0112

Ayeh, J. K. (2015). Travellers’ acceptance of consumer-generated media: An integrated model of technology acceptance and source credibility theories. *Computers in Human Behavior, 48*, 173-180.

Bagozzi, R. P., & Yi, Y. (2012). Specification, evaluation, and interpretation of structural equation models. *Journal of the academy of marketing science, 40*(1), 8-34.

Barreda, A. A., Bilgihan, A., Nusair, K., & Okumus, F. (2015). Generating brand awareness in online social networks. *Computers in Human Behavior, 50*, 600-609.

Bilal, M., Jianqiu, Z., Dukhaykh, S., Fan, M., & Trunk, A. (2021). Understanding the Effects of eWOM Antecedents on Online Purchase Intention in China. *Information, 12*(5), 192.

Bonn, M. A., Kim, W. G., Kang, S., & Cho, M. (2016). Purchasing wine online: The effects of social influence, perceived usefulness, perceived ease of use, and wine involvement. *Journal of Hospitality Marketing & Management, 25*(7), 841-869.

Bronner, F., & De Hoog, R. (2011). Vacationers and eWOM: who posts, and why, where, and what? *Journal of travel research, 50*(1), 15-26.

Chen, C.-C., & Chang, Y.-C. (2018). What drives purchase intention on Airbnb? Perspectives of consumer reviews, information quality, and media richness. *Telematics and Informatics, 35*(5), 1512-1523.

Chen, X., Tao, D., & Zhou, Z. (2019). Factors affecting reposting behaviour using a mobile phone-based user-generated-content online community application among Chinese young adults. *Behaviour & Information Technology, 38*(2), 120-131.

Cheng, X., & Zhou, M. (2010). *Study on effect of eWOM: A literature review and suggestions for future research.* Paper presented at the 2010 International conference on management and service science.

Cheung, C. M., Lee, M. K., & Rabjohn, N. (2008). The impact of electronic word‐of‐mouth: The adoption of online opinions in online customer communities. *Internet research, 146*, 931-944.

Cheung, C. M., & Thadani, D. R. (2012). The impact of electronic word-of-mouth communication: A literature analysis and integrative model. *Decision support systems, 54*(1), 461-470.

Cho, Y. C., & Sagynov, E. (2015). Exploring factors that affect usefulness, ease of use, trust, and purchase intention in the online environment. *International Journal of Management & Information Systems (IJMIS), 19*(1), 21-36.

Choi, Y. (2021). A Study of the Antecedents of e-WOM in Social Commerce Platform. *International Journal of Service Science, Management, Engineering, and Technology (IJSSMET), 12*(1), 62-76.

Chua, E. L., Chiu, J. L., & Chiu, C. L. (2020). Factors influencing trust and behavioral intention to use Airbnb service innovation in three ASEAN countries. *Asia Pacific Journal of Innovation and Entrepreneurship, 14*(2), 175–188.

Collis, J., & Hussey, R. (2013). *Business research: A practical guide for undergraduate and postgraduate students*: Macmillan International Higher Education.

Coyle, J. R., & Thorson, E. (2001). The effects of progressive levels of interactivity and vividness in web marketing sites. *Journal of advertising, 30*(3), 65-77.

Dancer, H., Filieri, R., & Grundy, D. (2014). eWOM in online customer support communities: Key variables in information quality and source credibility. *Journal of Direct, Data and Digital Marketing Practice, 15*(4), 290-305.

Dang, V. T., Wang, J., & Vu, T. T. (2020). An integrated model of the younger generation’s online shopping behavior based on empirical evidence gathered from an emerging economy. *PloS one, 15*(5), e0232213.

Daowd, A., Hasan, R., Eldabi, T., Rafi-ul-Shan, P. M., Cao, D., & Kasemsarn, N. (2020). Factors affecting eWOM credibility, information adoption and purchase intention on Generation Y: a case from Thailand. *Journal of enterprise information management, 34*(3), 838-859.

Davis, F. D. (1989). Perceived usefulness, perceived ease of use, and user acceptance of information technology. *MIS quarterly*, 319-340.

de Luna, I. R., Liébana-Cabanillas, F., Sánchez-Fernández, J., & Munoz-Leiva, F. (2019). Mobile payment is not all the same: The adoption of mobile payment systems depending on the technology applied. *Technological Forecasting and Social Change, 146*, 931-944.

Doh, S.-J., & Hwang, J.-S. (2009). How consumers evaluate eWOM (electronic word-of-mouth) messages. *Cyberpsychology & behavior, 12*(2), 193-197.

Elwalda, A., Lü, K., & Ali, M. (2016). Perceived derived attributes of online customer reviews. *Computers in Human Behavior, 56*, 306-319.

Erkan, I., & Evans, C. (2016). The influence of eWOM in social media on consumers’ purchase intentions: An extended approach to information adoption. *Computers in Human Behavior, 61*, 47-55.

Erkan, I., & Evans, C. (2018). Social media or shopping websites? The influence of eWOM on consumers’ online purchase intentions. *Journal of Marketing Communications, 24*(6), 617-632.

Eti, I. A., Horaira, M. A., & Bari, M. M. (2021). Power and stimulus of social media marketing on consumer purchase intention in Bangladesh during the COVID-19. International *Journal of Research in Business and Social Science*, 10(1), 28–37.

Etikan, I., Musa, S. A., & Alkassim, R. S. (2016). Comparison of convenience sampling and purposive sampling. *American journal of theoretical and applied statistics, 5*(1), 1-4.

Fang, Y. H. (2014). Beyond the credibility of electronic word of mouth: Exploring eWOM adoption on social networking sites from affective and curiosity perspectives. *International journal of electronic commerce, 18*(3), 67-102.

Filieri, R., Acikgoz, F., Ndou, V., & Dwivedi, Y. (2020). Is TripAdvisor still relevant? The influence of review credibility, review usefulness, and ease of use on consumers’ continuance intention. *International Journal of Contemporary Hospitality Management, 33*(1), 199-223.

Filieri, R., & McLeay, F. (2014). E-WOM and accommodation: An analysis of the factors that influence travelers’ adoption of information from online reviews. *Journal of travel research, 53*(1), 44-57.

Fornell, C., & Larcker, D. F. (1981). Evaluating structural equation models with unobservable variables and measurement error. *Journal of marketing research, 18*(1), 39-50.

Gray, D. E. (2013). *Doing Research in the Real World*: Sage Publishing Ltd.

Gvili, Y., & Levy, S. (2018). Consumer engagement with eWOM on social media: The role of social capital. *Online Information Review, 42*(4), 482-505.

Hafez, M. (2021). The Role of Social Media Marketing on Overall Brand Equity in the Telecommunication Sector in Bangladesh: A Moderated Mediation Model of Brand Love and Value Co-Creation. *International Journal of Asian Business and Information Management*, 12(3), 1-15.

Hair Jr, J. F., Hult, G. T. M., Ringle, C. M., & Sarstedt, M. (2021). *A primer on partial least squares structural equation modeling (PLS-SEM)*: Sage publications.

Hajiheydari, N., & Ashkani, M. (2018). Mobile application user behavior in the developing countries: A survey in Iran. *Information Systems, 77*, 22-33.

Hajli, N. (2018). Ethical environment in the online communities by information credibility: a social media perspective. *Journal of Business Ethics, 149*(4), 799-810.

Hanzaee, K. H., & Esmaeilpour, F. (2017). Effect of restaurant reward programs on customers’ loyalty: evidence from Iran. *Journal of Islamic Marketing, 8*(1), 140-155.

Hennig-Thurau, T., Gwinner, K. P., Walsh, G., & Gremler, D. D. (2004). Electronic word-of-mouth via consumer-opinion platforms: what motivates consumers to articulate themselves on the internet? *Journal of interactive marketing, 18*(1), 38-52.

Henseler, J., Ringle, C. M., & Sarstedt, M. (2015). A new criterion for assessing discriminant validity in variance-based structural equation modeling. *Journal of the academy of marketing science, 43*(1), 115-135.

Herrero, A., & Martínez, P. (2021). Determinants of electronic word-of-mouth on social networking sites about negative news on CSR. *Journal of Business Ethics, 171*(3), 583-597.

Humida, T., Al Mamun, M. H., & Keikhosrokiani, P. (2021). Predicting behavioral intention to use e-learning system: A case-study in Begum Rokeya University, Rangpur, Bangladesh. *Education and Information Technologies*, 1-25.

Hussain, S., Ahmed, W., Jafar, R. M. S., Rabnawaz, A., & Jianzhou, Y. (2017). eWOM source credibility, perceived risk and food product customer’s information adoption. *Computers in Human Behavior, 66*, 96-102.

Israel, K., Zerres, C., & Tscheulin, D. K. (2019). Presenting hotels in virtual reality: does it influence the booking intention? *Journal of Hospitality and Tourism Technology, 10*(3), 473–493.

Jiang, G., Liu, F., Liu, W., Liu, S., Chen, Y., & Xu, D. (2021). Effects of information quality on information adoption on social media review platforms: Moderating role of perceived risk. *Data Science and Management, 1*(1), 13-22.

Kamal, S. A., Shafiq, M., & Kakria, P. (2020). Investigating acceptance of telemedicine services through an extended technology acceptance model (TAM). *Technology in Society, 60*, 101212.

Kang, J.-W., & Namkung, Y. (2019). The role of personalization on continuance intention in food service mobile apps: A privacy calculus perspective. *International Journal of Contemporary Hospitality Management, 31*(2), 734–752.

Khwaja, M. G., Mahmood, S., & Jusoh, A. (2020). Online information bombardment! How does eWOM on social media lead to consumer purchase intentions? *International Journal of Grid and Utility Computing, 11*(6), 857-867.

Kim, M. J., Chung, N., Lee, C.-K., & Preis, M. W. (2016). Dual-route of persuasive communications in mobile tourism shopping. *Telematics and Informatics, 33*(2), 293-308.

Knoll, J. (2016). Advertising in social media: a review of empirical evidence. *International journal of Advertising, 35*(2), 266-300.

Kucukusta, D., Law, R., Besbes, A., & Legohérel, P. (2015). Re-examining perceived usefulness and ease of use in online booking: The case of Hong Kong online users. *International Journal of Contemporary Hospitality Management, 27*(2), 185-198.

Kumar, V. R., Lall, A., & Mane, T. (2017). Extending the TAM model: Intention of management students to use mobile banking: Evidence from India. *Global Business Review, 18*(1), 238-249.

Lee, H., Kim, J. W., & Hackney, R. (2011). Knowledge hoarding and user acceptance of online discussion board systems in eLearning: A case study. *Computers in Human Behavior, 27*(4), 1431-1437.

Lee, S. W., Sung, H. J., & Jeon, H. M. (2019). Determinants of continuous intention on food delivery apps: extending UTAUT2 with information quality. *Sustainability, 11*(11), 3141.

Leong, C. M., Loi, A. M. W., & Woon, S. (2021). The influence of social media eWOM information on purchase intention. *Journal of Marketing Analytics*, 1-13.

Li, C. Y. (2013). Persuasive messages on information system acceptance: A theoretical extension of elaboration likelihood model and social influence theory. *Computers in Human Behavior, 29*(1), 264-275.

Liao, S.-H., Hu, D.-C., Chung, Y.-C., & Huang, A.-P. (2021). Risk and opportunity for online purchase intention–A moderated mediation model investigation. *Telematics and Informatics, 62*, 101621.

Liu, S. Q., Ozanne, M., & Mattila, A. S. (2018). Does expressing subjectivity in online reviews enhance persuasion? *Journal of Consumer Marketing, 35*(4), 403-413.

Matute, J., Polo-Redondo, Y., & Utrillas, A. (2016). The influence of EWOM characteristics on online repurchase intention: Mediating roles of trust and perceived usefulness. *Online Information Review*.

Moslehpour, M., Pham, V. K., Wong, W.-K., & Bilgiçli, İ. (2018). E-purchase intention of Taiwanese consumers: Sustainable mediation of perceived usefulness and perceived ease of use. *Sustainability, 10*(1), 234.

Musil, C. M., Jones, S. L., & Warner, C. D. (1998). Structural equation modeling and its relationship to multiple regression and factor analysis. *Research in Nursing & Health, 21*(3), 271-281.

Ngarmwongnoi, C., Oliveira, J. S., AbedRabbo, M., & Mousavi, S. (2020). The implications of eWOM adoption on the customer journey. *Journal of Consumer Marketing, 37*(7), 749-759.

Nonaka, I. (1994). A dynamic theory of organizational knowledge creation. *Organization science, 5*(1), 14-37.

Özkan, S., Bindusara, G., & Hackney, R. (2010). Facilitating the adoption of e‐payment systems: theoretical constructs and empirical analysis. *Journal of enterprise information management, 23*(3), 305-325.

Park, D.-H., Lee, J., & Han, I. (2007). The effect of online consumer reviews on consumer purchasing intention: The moderating role of involvement. *International journal of electronic commerce, 11*(4), 125-148.

Park, E., & Kim, K. J. (2014). An integrated adoption model of mobile cloud services: exploration of key determinants and extension of technology acceptance model. *Telematics and Informatics, 31*(3), 376-385.

Petty, R. E., & Cacioppo, J. T. (1986). The elaboration likelihood model of persuasion *Communication and persuasion* (pp. 1-24): Springer.

Prendergast, G., Ko, D., & Siu Yin, V. Y. (2010). Online word of mouth and consumer purchase intentions. *International journal of Advertising, 29*(5), 687-708.

Qashou, A. (2021). Influencing factors in M-learning adoption in higher education. *Education and information technologies, 26*(2), 1755-1785.

Riana, D., Hidayanto, A. N., Hadianti, S., & Napitupulu, D. (2021). Integrative Factors of E-Health Laboratory Adoption: A Case of Indonesia. *Future Internet, 13*(2), 26.

Rimadias, S., Herlambang, A., Muladi, A., & Dharma, H. (2021). The role of E-WOM in social media on purchase intention on the Shopee e-commerce platform in Indonesia *Synergizing Management, Technology and Innovation in Generating Sustainable and Competitive Business Growth* (pp. 93-98): Routledge.

Roca, J. C., Chiu, C.-M., & Martínez, F. J. (2006). Understanding e-learning continuance intention: An extension of the Technology Acceptance Model. *International Journal of human-computer studies, 64*(8), 683-696.

Roy, G., Datta, B., & Mukherjee, S. (2019). Role of electronic word-of-mouth content and valence in influencing online purchase behavior. *Journal of Marketing Communications, 25*(6), 661-684.

Sarker, P. M., & Khayer, A. (2021). Factors Affecting Bangladeshi Customers’ Intention to Adopt Social Shopping: A Partial Least Square (PLS) Approach. *BUFT Journal of Business & Economics*, 2, 115-133

Sardar, A., Manzoor, A., Shaikh, K. A., & Ali, L. (2021). An Empirical Examination of the Impact of eWom Information on Young Consumers’ Online Purchase Intention: Mediating Role of eWom Information Adoption. *SAGE Open*, 11(4), 1-12.

Savolainen, R. (2011). Judging the quality and credibility of information in Internet discussion forums. *Journal of the American Society for Information Science and Technology, 62*(7), 1243-1256.

See-To, E. W., & Ho, K. K. (2014). Value co-creation and purchase intention in social network sites: The role of electronic Word-of-Mouth and trust–A theoretical analysis. *Computers in Human Behavior, 31*, 182-189.

Šerić, M., & Praničević, D. G. (2018). Consumer-generated reviews on social media and brand relationship outcomes in the fast-food chain industry. *Journal of Hospitality Marketing & Management, 27*(2), 218-238.

Shen, X. L., Cheung, C. M., & Lee, M. K. (2013). What leads students to adopt information from W ikipedia? An empirical investigation into the role of trust and information usefulness. *British Journal of Educational Technology, 44*(3), 502-517.

Shen, X. L., Zhang, K. Z., & Zhao, S. J. (2016). Herd behavior in consumers’ adoption of online reviews. *Journal of the Association for Information Science and Technology, 67*(11), 2754-2765.

Shu, M., & Scott, N. (2014). Influence of social media on Chinese students’ choice of an overseas study destination: An information adoption model perspective. *Journal of Travel & Tourism Marketing, 31*(2), 286-302.

Song, B. L., Liew, C. Y., Sia, J. Y., & Gopal, K. (2021). Electronic word-of-mouth in travel social networking sites and young consumers’ purchase intentions: an extended information adoption model. *Young Consumers*. 22(4), 521-538.

Song, S., Zhang, Y., & Yu, B. (2020). Interventions to support consumer evaluation of online health information credibility: A scoping review. *International Journal of Medical Informatics*, 104321.

Spatar, D., Kok, O., Basoglu, N., & Daim, T. (2019). Adoption factors of electronic health record systems. *Technology in Society, 58*, 101144.

Sussman, S. W., & Siegal, W. S. (2003). Informational influence in organizations: An integrated approach to knowledge adoption. *Information systems research, 14*(1), 47-65.

Sweeney, J. C., Soutar, G. N., & Mazzarol, T. (2012). Word of mouth: measuring the power of individual messages. *European Journal of Marketing, 46*(1/2), 237-257.

Tarhini, A., Arachchilage, N. A. G., & Abbasi, M. S. (2015). A critical review of theories and models of technology adoption and acceptance in information system research. *International Journal of Technology Diffusion (IJTD), 6*(4), 58-77.

Tien, D. H., Rivas, A. A. A., & Liao, Y.-K. (2019). Examining the influence of customer-to-customer electronic word-of-mouth on purchase intention in social networking sites. *Asia Pacific Management Review, 24*(3), 238-249.

Ventre, I., & Kolbe, D. (2020). The impact of perceived usefulness of online reviews, trust and perceived risk on online purchase intention in emerging markets: A Mexican perspective. *Journal of International Consumer Marketing, 32*(4), 287-299.

Verhellen, Y., Dens, N., & De Pelsmacker, P. (2013). Consumer responses to brands placed in youtube movies: The effect of prominence and endorser expertise. *Journal of Electronic Commerce Research, 14*(4), 287-303.

Wang, S., Cunningham, N. R., & Eastin, M. S. (2015). The impact of eWOM message characteristics on the perceived effectiveness of online consumer reviews. *Journal of Interactive Advertising, 15*(2), 151-159.

Watts, S., & Wyner, G. (2011). Designing and theorizing the adoption of mobile technology‐mediated ethical consumption tools. *Information Technology & People, 24*(3), 257-280.

Yang, F. X. (2017). Effects of restaurant satisfaction and knowledge sharing motivation on eWOM intentions: the moderating role of technology acceptance factors. *Journal of Hospitality & Tourism Research, 41*(1), 93-127.

Yeap, J. A., Ignatius, J., & Ramayah, T. (2014). Determining consumers’ most preferred eWOM platform for movie reviews: A fuzzy analytic hierarchy process approach. *Computers in Human Behavior, 31*, 250-258.

Yin, C., Sun, Y., Fang, Y., & Lim, K. (2018). Exploring the dual-role of cognitive heuristics and the moderating effect of gender in microblog information credibility evaluation. *Information Technology & People, 31*(3).

Yusuf, A. S., & Busalim, A. H. (2018). Influence of e-WOM engagement on consumer purchase intention in social commerce. *Journal of Services Marketing, 32*(4), 493-504.

Zha, X., Yang, H., Yan, Y., Liu, K., & Huang, C. (2018). Exploring the effect of social media information quality, source credibility and reputation on informational fit-to-task: Moderating role of focused immersion. *Computers in Human Behavior, 79*, 227-237.

Zhang, J., Ito, N., Wu, W., & Li, Z. (2017). “Don’t Let Me Think!” Chinese Adoption of Travel Information on Social Media: Moderating Effects of Self-disclosure *Information and Communication Technologies in Tourism 2017* (pp. 639-653): Springer.

Zhao, J., Wang, J., Fang, S., & Jin, P. (2018). Towards sustainable development of online communities in the big data era: A study of the causes and possible consequence of voting on user reviews. *Sustainability, 10*(9), 3156.

Zheng, Y., Zhao, K., & Stylianou, A. (2013). The impacts of information quality and system quality on users’ continuance intention in information-exchange virtual communities: An empirical investigation. *Decision support systems, 56*, 513-524.

Zhou, S., Yan, Q., Yan, M., & Shen, C. (2020). Tourists’ emotional changes and eWOM behavior on social media and integrated tourism websites. *International Journal of Tourism Research, 22*(3), 336-350.

Zhu, D. H., Chang, Y. P., & Luo, J. J. (2016). Understanding the influence of C2C communication on purchase decision in online communities from a perspective of information adoption model. *Telematics and Informatics, 33*(1), 8-16.
