# Supplementary material for: The interplay between eWOM information and purchase intention on social media: Through the lens of IAM and TAM theory
Source: PLoS One. 2022 Sep 6;17(9):e0272926. doi: 10.1371/journal.pone.0272926 (PMC9447927; doi:10.1371/journal.pone.0272926)
Supplement: Attachment — Submitted filename: Responses to Reviewers_PONE-D-21-35683R2.docx [file pone.0272926.s006.docx]

**Responses to Reviewers (PONE-D-21-35683R2**)

“The Interplay between eWOM Information and Purchase Intention on Social Media: Through the Lens of IAM and TAM Theory”

19 July 2022

Respected Reviewer,

Kindly find the below point to point responses to all reviewers’ comments

**Reviewer 2:**

| No. | Comments of Reviewer-02 | Our Responses |
| --- | --- | --- |
| 1. | If the authors have adequately addressed your comments raised in a previous round of review and you feel that this manuscript is now acceptable for publication, you may indicate that here to bypass the “Comments to the Author” section, enter your conflict of interest statement in the “Confidential to Editor” section, and submit your "Accept" recommendation.  Reviewer-2’s comment: (No Response) | Thank you. |
| 2. | Is the manuscript technically sound, and do the data support the conclusions?  The manuscript must describe a technically sound piece of scientific research with data that supports the conclusions. Experiments must have been conducted rigorously, with appropriate controls, replication, and sample sizes. The conclusions must be drawn appropriately based on the data presented.  Reviewer-2’s comment: Yes | Thank you so much for your positive acceptance. |
| 3. | Has the statistical analysis been performed appropriately and rigorously?  Reviewer-2’s comment: Yes | Thank you so much for your positive compliment. |
| 4. | Have the authors made all data underlying the findings in their manuscript fully available?  The PLOS Data policy requires authors to make all data underlying the findings described in their manuscript fully available without restriction, with rare exception (please refer to the Data Availability Statement in the manuscript PDF file). The data should be provided as part of the manuscript or its supporting information, or deposited to a public repository. For example, in addition to summary statistics, the data points behind means, medians and variance measures should be available. If there are restrictions on publicly sharing data—e.g. participant privacy or use of data from a third party—those must be specified.  Reviewer-2’s comment: Yes | Thank you so much for your positive compliment.. |
| 5. | Is the manuscript presented in an intelligible fashion and written in standard English?  PLOS ONE does not copyedit accepted manuscripts, so the language in submitted articles must be clear, correct, and unambiguous. Any typographical or grammatical errors should be corrected at revision, so please note any specific errors here.  Reviewer-2’s comment: Yes | Thank you so much for your positive compliment. |
| 6. | Review Comments to the Author  Please use the space provided to explain your answers to the questions above. You may also include additional comments for the author, including concerns about dual publication, research ethics, or publication ethics. (Please upload your review as an attachment if it exceeds 20,000 characters)\  Reviewer #2: There are still some mistakes as pointed out in the attached text attached. Some suggestions been provided too. Is up to you to decide whether you want to correct or not. I can't say I am 100% correct. Is up to you and up to Plos One to accept your paper. I don't have any other concern of recommending the acceptance of your paper. Whatever written is a reflection of your work. You may contact me if you want at [lswong@utar.edu.my](mailto:lswong@utar.edu.my) | Thank you so much for your kind feedback in the attached manuscript. We have tried our best to correct the grammatical issues in the manuscript (kindly refer to yellow marked sections). |

**NOTE:** This study was supported by the School of Management, Jiujiang University and Center for Entreprenureship & Innovation Research Society (grant number: 43120227). The funders have no role in study design, data collection and analysis, decision to publish, or preparation of the manuscript. In regard to data availability statement, we’ve provided all necessary data files with the manuscript (please refer to 1. Raw data file_csv file, 2. Measurement model_xlsx file, 3. Structural model__xlsx file).

Kind Regards,

The Corresponding Author:

*Ahmed Al Asheq*

1) PhD Student

School of Business

Maynooth University, Ireland.

Email: ahmed.asheq.2022@mumail.ie

2) Lecturer

Department of Business Administration

World University of Bangladesh (WUB), Bangladesh.

Email: aasheq@business.wub.edu.bd

**Other Authors:**

Dr. Md. Atikur Rahaman (email: atik@jju.edu.cn)

H. M. Kamrul Hassan (email: kamrul@cu.ac.bd)

K. M. Anwarul Islam (email: anwarul@themillenniumuniversity.edu.bd)
